# Synaptic Plasticity Dysfunctions in the Pathophysiology of 22q11 Deletion Syndrome: Is There a Role for Astrocytes?

**DOI:** 10.3390/ijms23084412

**Published:** 2022-04-16

**Authors:** Eva Cristina de Oliveira Figueiredo, Bianca Maria Bondiolotti, Anthony Laugeray, Paola Bezzi

**Affiliations:** 1 Department of Fundamental Neurosciences, University of Lausanne, 1005 Lausanne, Switzerland; evacristina.deoliveirafigueiredo@unil.ch (E.C.d.O.F.); biancambondiolotti@gmail.com (B.M.B.); anthony.laugeray@unil.ch (A.L.); 2 Department of Pharmacology and Physiology, University of Rome Sapienza, 00185 Rome, Italy

**Keywords:** 22q11 deletion syndrome, synaptic plasticity, synapses, mitochondria, astrocytes

## Abstract

The 22q11 deletion syndrome (DS) is the most common microdeletion syndrome in humans and gives a high probability of developing psychiatric disorders. Synaptic and neuronal malfunctions appear to be at the core of the symptoms presented by patients. In fact, it has long been suggested that the behavioural and cognitive impairments observed in 22q11DS are probably due to alterations in the mechanisms regulating synaptic function and plasticity. Often, synaptic changes are related to structural and functional changes observed in patients with cognitive dysfunctions, therefore suggesting that synaptic plasticity has a crucial role in the pathophysiology of the syndrome. Most interestingly, among the genes deleted in 22q11DS, six encode for mitochondrial proteins that, in mouse models, are highly expressed just after birth, when active synaptogenesis occurs, therefore indicating that mitochondrial processes are strictly related to synapse formation and maintenance of a correct synaptic signalling. Because correct synaptic functioning, not only requires correct neuronal function and metabolism, but also needs the active contribution of astrocytes, we summarize in this review recent studies showing the involvement of synaptic plasticity in the pathophysiology of 22q11DS and we discuss the relevance of mitochondria in these processes and the possible involvement of astrocytes.

## 1. Introduction

The 22q11 deletion syndrome (DS), also known as DiGeorge or velocardiofacial syndrome, which occurs in one out of 4000 live births is caused by a hemizygous microdeletion in the long arm of chromosome 22, the most frequent of which are a three megabase (Mb) deletion that affects ~60 genes and a 1.5 Mb deletion affecting 35 genes [1,2]. It is thought that the deletion is caused by a non-allelic homologous recombination due to the presence of a low copy number of repeats flanking the region that predispose to ectopic recombination during meiosis [3]. Most affected subjects (85%) have a 3 Mb deletion encompassing approximately 30 contiguous genes, but ~15% have smaller atypical deletions of 1.5 Mb [4] that seem to contain all the genes necessary for the development of the syndrome [5] and an increased risk of psychosis [6,7].

The syndromic nature of the disorder gives rise to various phenotypes that can involve different organs and tissues, but most patients (~76%) have congenital heart defects, palatal and renal abnormalities, characteristic craniofacial dysmorphisms and hypocalcaemia, and are affected by immune deficiency and learning difficulties that lead to major developmental delays (see [3] for the detailed pathophysiological mechanisms and a comprehensive review). The 22q11DS is also associated with a strikingly high risk of developing neuropsychiatric disorders: about 25–30% of patients develop affective psychosis or schizophrenia, thus making the syndrome one of the greatest risk factors for psychotic disorders identified so far [8,9]. The 22q11.2 microdeletion accounts for up to 2% of all cases of schizophrenia and is the only known recurrent copy number variation (CNV) responsible for new cases of schizophrenia [4,7].

**Figure 1:**
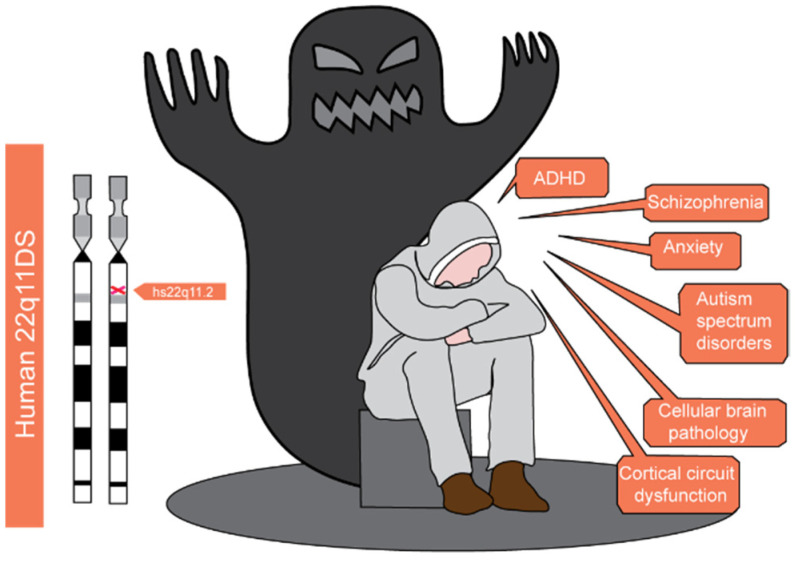
**Phenotypic features of 22q11.2 Deletion Syndrome (22q11DS).** The chromosomic location of the deletion (**left**) and the most common manifestations of the disease (**right**) are shown. Starting from childhood and early adolescence 22q11.2 subjects face a wide range of neuropsychiatric illnesses which include attention deficit disorder (ADHD), schizophrenia spectrum disorder, anxiety, and autism spectrum disorders. A baseline cellular brain pathology and the consequent cortical circuit dysfunction are at the root of these alterations.

Furthermore, the prevalence of other neuropsychiatric disorders (Figure 1) such as autism spectrum disorder (ASD), anxiety, and attention deficit hyperactivity disorder (ADHD) [10,11] is considerably higher in 22q11DS patients than in the general population [12,13,14]. ADHD and anxiety disorders are the most frequent diagnoses during childhood, whereas the rates of psychosis and mood disorders increase dramatically during adolescence and young adulthood. Although the average age of 22q11DS patients at the time of the onset of overt psychotic disorders is 19–26 years [15], earlier manifestations of psychotic-like symptoms characterise almost one-third of adolescents and ~17% of pre-adolescent children [12,16], thus suggesting that the severity of the psychotic symptoms progressively worsens.

The 22q11.2DS is associated with a wide range of cognitive impairments [13,17]. Patients generally have poor non-verbal, numerical, and spatio-temporal skills [18] and their impaired attention system [19] prevents them from distinguishing relevant from irrelevant information and fails to inhibit the impulsive responses that interfere with the brain’s ability to focus on goal-relevant thoughts. Furthermore, those with psychotic symptoms are associated with cognitive deficits and a decline in academic performance. It has been reported that measures of executive functions such as working memory and sustained attention reveal greater deficits in 22q11.2DS patients with schizophrenia than in those without [20,21,22], and longitudinal studies following adolescents with 22q11.2DS into adulthood have shown that the IQ of patients with psychotic disorders is lower than in those without [23].

Studies of 22q11DS patients and mouse models (see next paragraph) have indicated that the cognitive deficits may be due to deficits in synaptic plasticity in the cortical [24,25] and hippocampal circuits that are known to be involved in various aspects of planning, working memory, rule-based learning, attention, and emotional regulation [26,27]. Plasticity refers to the ability of neural activity to modify the neural circuit functions generated by an experience (such as subsequent thoughts, feelings, and behaviour). “Synaptic plasticity” specifically refers to activity-dependent modifications of the strength or efficacy of synaptic transmission at pre-existing synapses. For more than a century, it has been suggested that this mechanism plays a central role in the capacity of the brain to incorporate transient experiences into persistent memory traces [28,29] and it is therefore also thought to be critically involved in the early development of neural circuits [30]. Many forms and mechanisms of short- and long-term synaptic plasticity have been described (including paired-pulse facilitation and depression, facilitation and depression following trains of stimuli, and the modulation of transmission by pre-synaptic receptors), and the temporal domains of such changes range from milliseconds to hours, days, and presumably even longer [30]. Over the last ten years, several studies have indicated that deficits in synaptic plasticity may contribute to the cognitive deficits associated with many neurodevelopmental disorders, including 22q11DS [6,31,32,33,34,35,36], which suggests that elucidating the mechanisms underlying synaptic plasticity may be a crucial step in improving our understanding of the disorders themselves. 

Furthermore, there is increasing evidence suggesting that astrocytes may be involved in some forms of short-term and long-term plasticity [37,38,39,40,41,42] by changing their synaptic coverage, controlling the clearance of neurotransmitters, or by releasing neuroactive substances (i.e., gliotransmitters, cytokines, and chemokines) that can directly affect synaptic efficacy [37,39,42].

## 2. Synaptic Plasticity and the Pathophysiology of 22q11DS

After having exposed how behavioural and cognitive deficits are an integral part of the pathogeny of the 22q11DS and highly evolve throughout a patients’ life, we now focus on details regarding the extent to which synaptic plasticity alterations are crucially involved in the occurrence of cognitive defects during the disease. With this aim, we first emphasize how 22q11 deleted genes are likely to affect synaptic plasticity processes in animal models of the disease, and then, describe how mitochondrial defects and altered astrocytic development are closely entangled and may ultimately underlie cognitive deficits associated with synaptic plasticity changes. 

Over the last ten years, it has been suggested that behavioural and cognitive impairments underlying most neuropsychiatric diseases are probably due to alterations in the mechanisms regulating synaptic function and plasticity [43]. In the case of 22q11DS, many of the transcribed genes in the human minimally critical 1.5 MB and the larger 3 MB deleted region encode proteins involved in synaptic processes, and studies of animal models have confirmed the role of many 22q11 genes in regulating synaptic transmission and plasticity [4,35]. Furthermore, in comparison with controls, histological studies of cell-derived neurons (hiPSC cells and human organoids) from 22q11DS patients have confirmed the synaptic deficit by revealing a reduction in dendritic arborisation and spines [6,32,36] as well as in synapse markers [32]. 

Neuronal cell dysmorphologies are common to many 22q11DS phenotypes (cardiovascular, craniofacial and limb malformations, and thymic dysplasia), which suggests that the heterozygous 1.5 or 3 MB deletion and the consequent reduction in 22q11 genes disrupt the development of various systems, including the brain. Similarly, the significant susceptibility of 22q11DS patients to the development of schizophrenia, ASD, ADHD, language delay, and other behavioural alterations [15,44], all of which are features of neurodevelopmental disorders [45,46,47], suggests that 22q11DS disrupts brain development as well as peripheral and visceral morphogenesis.

It has also been suggested that 22q11 genes may differentially operate at distinct times during development [48]: for example, a reduction in a subset of genes expressed at sites of mesenchymal/epithelial interaction may compromise early brain, face, heart, and limb morphogenesis; a reduction in the genes regulating the cell cycle may disrupt neurogenesis in the cerebral cortex; and a reduction in a distinct subset of mitochondrial genes may compromise post-natal astrocyte formation and maturation, dendrite and axon formation, and synaptogenesis. Such combined and/or recurrent dysfunctions in 22q11DS genes may disrupt the differentiation and maturation of brain cells from early embryonic to late post-natal development, and this may explain the variable behavioural pathology and dysmorphologies associated with 22q11DS. Neuronal cell dysmorphologies are also consistent with widespread reductions in the volume of, particularly, the hippocampus and pre-frontal and temporal cortices [49,50,51,52,53] and this suggests alterations in the functional connectivity of, particularly, fronto-limbic circuitry [54,55,56]. 

However, although these cellular and morphological observations suggesting the possible involvement of altered synaptic plasticity in 22q11DS do not explain the complex symptoms associated with 22q11DS pathology, circuit-level explanations may be a means of integrating the information coming from studies of patients and animal models. Animal studies indicate that altered synaptic function in hippocampal and cortical brain structures is crucially involved in behavioural and cognitive impairments observed in most neuropsychiatric diseases [43]. In line with this, many laboratories have developed complementary models that relate synaptic changes to the structural and functional changes observed in patients with behavioural and cognitive dysfunctions [4,57].

**Figure 2:**
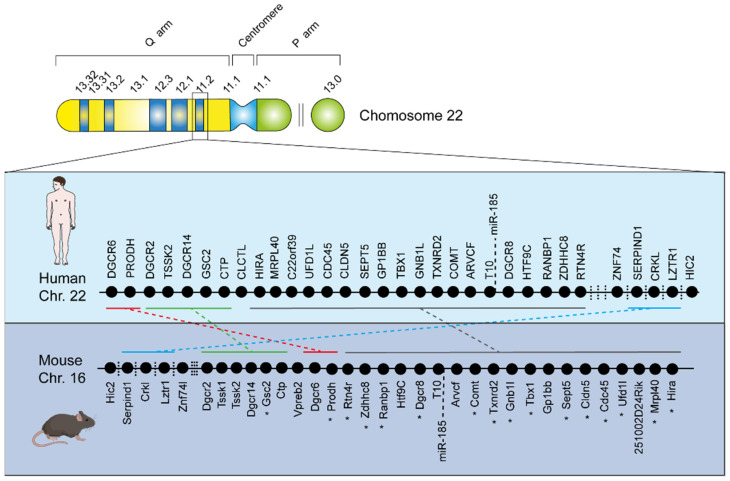
**Genetic background of 22q11 DS.** The ideogram of chromosome 22 is shown: there is a short (p) arm and long (q) arm, along with the centromere. The 22q11.2 deletion occurs in the q arm, as indicated by the box in the 22q11.2 band. The black dots represent the genes within the commonly deleted region of Chr. 22 in Humans (light blue box) and of the corresponding Chr. 16 in Mice (blue box). Dashed lines connecting groups of identical genes depict the important analogy between these genomic regions in Humans and in Mouse Models. Asterisks indicate mitochondrial genes. This characteristic allows the use of animal models to study molecular mechanisms that cannot be easily inferred working solely with human subjects.

The orthologous murine region of the human 22q11 locus lies on mouse chromosome 16, and all but one of the human genes in this region are represented, although they are organised in a different order [4,58]. Several mouse models carrying chromosomal deficiencies that are in synteny with the human 22q11.2 microdeletion have been generated [4,59,60], including Df1/^+^ and LgDel^+/−^ mice carrying a hemizygous deletion of 18 and 24 genes respectively in the 22q11DS-related region of mouse chromosome 16 [60,61,62] (Figure 2). Mouse models of 22q11DS are among the few animal models that replicate the abnormalities associated with the syndrome in humans: for example, they reveal cognitive deficits in the conditioned contextual fear paradigm, an assay that partially depends on the hippocampus and pre-frontal cortex (PFC) [6,63,64], and have shown abnormalities in corticogenesis and development of dendrites and dendritic spines in hippocampal and PFC pyramidal neurons [4,6,27,57,65,66], highlighting the importance of correct neuronal development. As previously said, cognitive deficits in 22q11 patients are mainly associated with a diagnosis of schizophrenia [67,68] and are now considered better predictors of disease progression than any other symptoms [23,69]. As in the case of schizophrenia, it is thought that 22q11DS-related cognitive symptoms (particularly deficits in spatial working memory) originate from the hippocampal and cortical regions involved in learning and memory [70,71]. Of note, these alterations have been shown to occur both in 22q11DS patients [14,51] and 22q11DS mouse models [26,27]. Furthermore, mouse models of 22q11DS reveal abnormal short- and long-term hippocampal synaptic plasticity [36,57,62,72,73], which is consistent with the idea that synaptic plasticity is a cellular mechanism of learning and memory [74,75].

Many studies have highlighted alterations in synaptic plasticity using animal models in which the genes forming the genetic basis of the psychiatric and cognitive symptoms of 22q11DS [76] have been deleted or mutated. For example, it has been found that a genetic variant of *ZDHHC8* (a gene predisposing to schizophrenia) [76,77] is causally related to a reduction in the strength of synaptic connections and alterations in the terminal arborisation of both cortical and hippocampal neurons [65]. *ZDHHC8* is a palmitoyltransferase that belongs to a 23-member family of enzymes sharing a conserved cysteine-rich signature catalytic domain (the DHHC domain) [78] and it has been shown that palmitoylation is a key reversible post-translational protein modification involved in protein trafficking and the regulation of various membrane and cytosolic proteins, especially in neurons [79]. The alterations in synaptic strength were accompanied by impaired mPFC-hippocampus connectivity and spatial working memory, one of the main cognitive features of early-stage schizophrenia [80]. Most of the synaptic dysfunctions observed in these *ZDHHC8*-deficient mice are also present in the Df(16)A^+^/^−^ mice model of 22q11DS carrying the 1.3 MB microdeletion in the mouse locus that is in synteny with the human 22q11.1 locus encompassing 27 genes [62]. 

It has also been found that the effects of ZDHHC8 are at least partially mediated by the Cdc42-dependent modulation of Akt/Gsk3b signalling, which is consistent with the increasingly supported association between dysregulated Akt/Cdc42 signalling dysregulation and schizophrenia in humans [81]. Furthermore, pre-clinical studies have demonstrated that impaired AKT signalling affects neuronal connectivity and neuromodulation within the PFC [82] and have identified AKT as a key signalling intermediary downstream of dopamine (DA) receptor 2 (DRD2), the most established target of the antipsychotic drugs used to treat schizophrenic patients. This strengthens the hypothesis of a relationship between 22q11 genes, impaired synaptic plasticity, and the cognitive and behavioural deficits observed in schizophrenia-expressing 22q11DS patients.

One of the genes deleted in most patients with 22q11DS is the DiGeorge critical region gene 8 (*DGCR8*), which encodes a crucial component of the micro-processing complex that contributes to the biogenesis of microRNA (miRNA). Approximately 22 nucleotides long, miRNAs regulate gene expression primarily by means of post-transcriptional gene silencing after binding to their target RNAs, and are involved in many biological processes including development, cell death, and cell metabolism [83,84]. Given the crucial role of 22q11 gene-mediated alterations in synaptic plasticity as the neural substrate underlying cognitive dysfunctions and increased risk of developing schizophrenia associated with 22q11 microdeletions, Fénelon et al. investigated the effect of *DGCR8* deficiency on the structure and function of cortical circuits by assessing their laminar organisation and the neuronal morphology and synaptic properties of layer 5 pyramidal neurons in the pre-frontal cortex of DGCR8^+^/^−^ mutant. They found that they had fewer cortical layer 2/4 neurons, and smaller spines in the basal dendrites of their layer 5 pyramidal neurons. In addition to these structural changes, field potential and whole-cell electrophysiological recordings of layer 5 of the PFC showed greater short-term synaptic depression in response to the stimulation of superficial cortical layers. As a key component of the micro-processing complex essential for miRNA production [85], it is likely that *DGCR8* significantly contributes to the miRNA dysregulation observed in the brain of Df(16)A^+^/^−^ mice [72]. 

It has been shown that a number of miRNAs regulate neuronal synaptic plasticity by means of the local synthesis of proteins at synaptic level [86]. In line with this, it has also been recently shown that the miRNA dysregulation due to *DGCR8* deficiency leads to deficits in various forms of prefrontal cortical synaptic plasticity, and is also likely to induce schizophrenia-related symptoms in Df(16)A^+^/^−^ mice [87]. Moreover, a reduction in the levels of *Mirta22* (an inhibitor of neuronal maturation whose expression is up-regulated in the brain of Df(16)A^+^/^−^ mice as a result of the hemizygosity of *DGCR8*) [88] rescued not only key schizophrenia-related cognitive and behavioural dysfunctions, but also abnormalities in PFC synaptic and structural plasticity.

Interestingly, Sivagnanasundram et al. found that the expression of 15 genes other than those involved in the initial deletion was significantly modified in the hippocampus of Df1/^+^ mice, [89]. Five of these genes (*Calm1*, *Pcdh8*, *Ube2d2*, *Uble1b*, and *Ywhaz*) are known to be involved in learning and memory processes and/or schizophrenia-like phenotypes frequently observed in 22q11DS patients. Calmodulin 1, which is encoded by *Calm1* and plays a key role in synaptic plasticity by regulating a large number of enzymes and proteins [90], a process that shapes long-term neuronal function (particularly long-term hippocampal potentiation and spatial learning) by modifying the phosphorylation/dephosphorylation balance of downstream target proteins [91,92], was downregulated by 27%. Furthermore, a reduction in the expression of *Pcdh8* and *Ywhaz* may also contribute to impairing synaptic plasticity in Df1/^+^ mice as the first encodes protocadherin 8, which modulate the synaptic plasticity that is essential to the process of learning and memory [93]. The second belongs to the 14-3-3 family and is thought to play a key role in neuronal differentiation, synaptic plasticity, and olfactory learning and memory [94]. Interestingly, it has been reported that disturbances in *Pcdh8* are closely associated with autistic-like symptoms [95], a clinical feature that is frequently observed in 22q11DS patients [96] and alterations in the expression of *Ywhaz* and other members of the 14-3-3 family have been identified in the PFC and cerebellum of schizophrenic patients [97]. These findings highlight the importance of impaired synaptic plasticity in the etiology of 22q11DS, particularly when it occurs within the PFC [98] or hippocampus [99] or along the PFC-hippocampus pathway [100].

## 3. Role of Mitochondrial Genes in the Dysfunctions of Synaptic Plasticity Associated with 22q11 DS

Intriguingly, nine of the many genes deleted in 22q11DS can induce mitochondrial homeostasis disorders (*COMT*, *UFD1L*, *DGCR8*, *MRPL40*, *PRODH*, *SLC25A1*, *TXNRD2*, *T10*, and *ZDHHC8*), but the first three may have no more than an indirect effect on mitochondrial function, and only the last six encode mitochondrial proteins [101]. These six genes are maximally expressed in mouse brain shortly after birth at a time of active synaptogenesis, which suggests that well-functioning mitochondrial processes may be intimately related to correct synapse formation. If this is so, alterations in mitochondrial metabolism during the early phases of brain development may pave the way for impaired neuronal metabolism or synaptic signalling, and thus partially explain the higher incidence of developmental and behavioural deficits in 22q11 patients. This review does not intend to cover precisely mitochondrial functions through brain development but, rather, to point out how deletion of the above-mentioned genes is causally related to cognitive deficits induced by synaptic plasticity disruption. In line with this, a recent study demonstrated a close correlation between the age-dependent decline in the working memory of monkeys and an increased prevalence of doughnut-shaped mitochondria in PFC presynaptic boutons [102] due to oxidative stress [103]. It is also worth noting that, of the six genes mentioned above, *TXNRD2* encodes the mitochondrial enzyme thioredoxin-reductase 2, which is of paramount importance for the mitochondrial scavenging of reactive oxygen species (ROS) [104]. Another recent study found that specific ROS accumulation in PFC layer 2-3 projecting neurons due to *TXNRD2* haploinsufficiency is not only causally related to alterations in local dendritic branching, but also to cognitive deficits measured during a touchscreen-mediated visual discrimination/reversal task [57]. It was also found that treating LgDel mouse models from birth to weaning with maternal drinking water containing the antioxidant N-acetylcysteine completely restored working memory in young adult mice, thus highlighting the central role of mitochondrial ROS-mediated stress in the occurrence of cognitive deficits in this 22q11DS mice model. 

In the same year, Gokhale et al. published a study showing an association between proteome changes in the SLC25A1-SLC25A4 mitochondrial interactome and synapse function in the Df(16)A^+^/^−^ mice model of 22q11DS [105]. *SLC25A1* encodes a mitochondrial citrate transport protein that allows exchanges of small metabolites between the mitochondrial matrix and the cytosol [106] and it was shown that its knockdown in zebrafish induced mitochondria depletion and proliferation defects, two phenotypes frequently described in 22q11DS patients [106]. More recently, another study demonstrated that the deletion of human *SLC25A1* compromises the integrity of the mitochondrial ribosome and down-regulates the expression of multiple ribosome subunits, including MRPL40 [107], which disrupts short-term synaptic plasticity and working memory by dysregulating mitochondrial calcium [73]. *MRPL40* encodes the mitochondrial ribosomal protein L40, a protein of the large subunit of the mitochondrial ribosome whose function is necessary for mitochondrial protein synthesis in eukaryotes [108]. As previously mentioned, *ZDHHC8* encodes a mitochondrial palmitoyl transferase enzyme that is involved in regulating the cell localisation of target proteins [109] and a reduction in its expression reduces the strength of synaptic connections and impairs terminal arborisation in the hippocampus-PFC circuit of a mice model of 22q11DS [65].

Lastly, it has been found that dysregulation of the *PRODH* gene, which encodes the mitochondrial enzyme proline dehydrogenase, is associated with the occurrence of schizophrenia-like phenotypes in humans [110] and animal models, probably because of alterations in glutamatergic and dopaminergic transmission, especially in the PFC and hippocampus [66]. Interestingly, *PRODH* and *ZDHHC8* both have single nucleotide polymorphisms (SNPs) that are closely associated with 22q11DS-related schizophrenia-like symptoms [111]. It has also been reported that the *T10* gene (also known as TANGO2), which encodes a member of the transport and Golgi organisation family that plays a role in endoplasmic reticulum (ER) secretory protein loading [112], has associated SNPs but at a less robust level [111].

Taken together, these findings not only underline the crucial role of mitochondrial alterations in the etiology of cognitive impairments observed in 22q11DS patients, but also indicate that these alterations are likely to be due to dysfunctions in synaptic processes, which is in line with the fact that two major mitochondrial functions (energy supply and calcium buffering) are proven regulators of synaptic plasticity [113]. In connection with this, Earls et al. showed that the dysregulation of calcium dynamics in the presynaptic terminals of CA3 neurons in Df1/^+^ mice alters long-term potentiation at excitatory synapses [36]. They also showed that these changes are due to the up regulation of sarcoendoplasmic reticulum calcium (Ca^2+^) ATPase (SERCA2), which is responsible for loading Ca^2+^ into the ER, and that the resulting increase in ER calcium load triggers enhanced neurotransmitter release and increases long term potentiation (LTP) levels [114]; furthermore, the depletion of calcium stores induced by a SERCA2 inhibitor fully rescues synaptic phenotypes observed in Df1/^+^ mice [36]. Interestingly, it has been found that SERCA2 levels are increased in brains of patients with idiopathic schizophrenia, thus providing a direct link between the processes described above and 22q11Ds-related psychiatric deficits.

## 4. Astrocytes Can Modulate Synaptic Plasticity: Do They Play a Role in the Pathophysiology of 22q11DS?

As reviewed so far, altered synaptic plasticity through mitochondrial deficits seems to be strongly involved in cognitive symptoms reported in 22q11DS patients. Even though it has long been considered that synapses have the inherent property of plasticity, a property based on mechanistic changes occurring within neurons [115], neurons do not function in isolation but belong to elaborate glial networks in which they are intimately associated with astrocytes [116]. A growing body of evidence suggests that correct synaptic functioning may involve the active participation of astrocytes [116,117,118]. It is possible that astrocytes may also be at least partially responsible for the regulation of synaptic plasticity [119,120,121]. Therefore, in the next sections of this review, we focus on how developing astrocytes may be involved in 22q11DS related synaptic plasticity and cognitive deficits through changes in dopamine and mitochondrial homeostasis.

Astrocytes have functional and structural domains that are estimated to be in contact with 200–600 dendrites and about 10^5^ synapses [122,123] and their extensive contacts with synaptic sites ensure strict control of local ions [124], pH homeostasis [125], the delivery of metabolic substrates to neurons [126,127,128,129], control of the microvasculature [130], and modulation of synaptic activity and plasticity by releasing neuroactive substances [42,131,132,133,134,135,136,137,138]. Perisynaptic astrocytes also express many transporters of amino acid neurotransmitters, including glutamate and GABA, and remove neurotransmitters to maintain transmitter homeostasis [139], thus assuring the rapid and efficient control of the speed and extent of neurotransmitter clearance, a mechanism involved in synaptic plasticity [140,141].

Recent studies showed that astrocytes also remove monoamine neurotransmitters, and various research groups demonstrated that cultured astrocytes and astrocytoma transport monoamines such as serotonin, dopamine, norepinephrine, and histamine [142,143,144,145]. Transcriptome and immunohistochemistry analyses confirmed the presence of monoamine transporters in in vivo astroglial cells, including organic cation transporter 3 (OCT3) [145,146,147,148,149,150] and plasma membrane monoamine transporter (PMAT) [150]. OCT3 and PMAT form part of the uptake system of low-affinity (higher Km values) and high-capacity transporters (higher Vmax values) of monoamines such as serotonin and dopamine regardless of extra-cellular Na^+^/Cl^−^, and recent studies indicated the critical role of low-affinity transporters in in vivo serotonin and dopamine clearance [151,152], although the relative importance of the two uptake systems is still unknown. 

It was recently suggested that the taking up of dopamine by the astroglial OCT3 and PMAT transporters in the developing PFC indicates their involvement in controlling dopamine homeostasis [150]. The PFC is different from other dopaminergic brain areas such as the striatum insofar as it expresses much lower levels of the high-affinity dopamine transporter (DAT) and dopamine uptake by DAT plays a marginal role in clearing extra-cellular dopamine levels [153]. Studies of the mechanisms regulating dopamine homeostasis in the PFC showed that dopamine clearance in the presence of low DAT levels depends on secondary mechanisms such as the metabolic activity of monoamine oxidase B (MAOB) and cathecol-o-methyltransferase (COMT), and uptake of the norepinephrine transporter [154,155,156,157]. 

Astrocytes in the developing PFC are equipped to control dopamine homeostasis as those expressing OCT3/PMAT and MAOB/COMT contain vesicular monoamine transporter 2 (VMAT2), which acts in concert with OCT3 and MAOB to provide effective control of the metabolic capacity of dopamine [150]. The dopamine entering via OCT3/PMAT transporters accumulates in VMAT2-positive intra-cellular organelles (i.e., endosomes or lysosomes), which are highly dynamic in terms of fusions and fissions and passively leak dopamine into the cytoplasm, where it is degraded by metabolic enzymes. This leakage seems to be a crucial step in the metabolism of catecholamines (including dopamine) in various cells including neurons [158]. In the absence of the VMAT2-dependent control of dopamine storage in astrocytes, the dopamine taken up by plasma membrane transporters is immediately metabolised, which triggers the aberrant uptake of the transporters and a consequently significant decrease in the extra-cellular dopamine levels [150] whereas, by expressing the determinants of dopamine control, astrocytes maintain the correct equilibrium of extra-cellular dopamine levels. In line with the particular nature of the PFC, this mechanism seems to be quite PFC-specific as VMAT2 is highly expressed by astrocytes located in the frontal and pre-frontal cortical areas, but absent from those in other cortical areas such as the visual cortex [150]. Interestingly, when this astroglial mechanism is perturbed (for example, in the absence of VMAT2), the concomitant decrease in extra-cellular dopamine levels may have significant effects on synaptic plasticity [150] as it triggers the accelerated spine maturation of pyramidal neurons in layer 5 of the PFC, increases the frequency of miniature excitatory postsynaptic currents (mEPSCs), and compromises LTP. Furthermore, the effect on synaptic strength is consistent with accelerated synaptic development, and this suggests that appropriate dopamine levels act as a developmental repressor via the activation of pre-synaptic D2 receptors [150]. In the absence of VMAT2, the alteration in synaptic plasticity is also accompanied by defects in executive cognitive functions, one of the hallmarks of schizophrenia and 22q11DS. The fact that the deletion of VMAT2 and subsequent decrease in dopamine causes excessive neuronal excitation, strongly suggests a neural network that is resistant to experience-dependent refinement and therefore prone to cognitive and behavioural deficits.

Is it possible that these mechanisms are also involved in the pathophysiology of the cognitive deficits associated with 22q11? The *COMT* gene encoding the cytoplasmic COMT enzyme, which (in addition to mitochondrially located MAOB) acts as a key degrader of dopamine, is contained within the critical 1.5 Mb 22q11 DS microdeletion. The dopamine hypothesis of schizophrenia, which has greatly influenced research into the mechanisms underlying the onset of psychosis, suggests that *COMT* is an attractive candidate gene underlying susceptibility to schizophrenia [159,160,161,162], and studies of *Comt*^−^/^−^ mice have demonstrated the ability of the COMT enzyme to control the extra-cellular clearance of PFC dopamine and influence cognitive functions such as working memory [6,163,164]. It can therefore be argued that reduced COMT activity may slow extra-cellular clearance and lead to the aberrant tonic stimulation of dopamine receptors in the PFC, a situation that has been associated with perseverative behaviour and inflexible cognitive performances [165,166] which is a hallmark of the cognitive deficits associated with schizophrenia [166]. 

When considering the role of *COMT* in the pathophysiology of 22q11DS-related cognitive dysfunctions, it is necessary to see *COMT* in the context of an epitastic interaction with the *PRODH* gene, which is also within the 22q11 microdeletion and encodes mitochondrially expressed proline dehydrogenase (PRODH). As previously mentioned, the PRODH enzyme is the rate-limiting enzyme in proline degradation [167] and the homozygous deletion of *PRODH* is associated with hyperprolinemia, which significantly increases proline levels in the brain and leads to significant neuropsychiatric dysfunctions [6,167]. Studies of mouse models and 22q11DS patients have provided evidence supporting the idea of an epistatic interaction between *COMT* and *PRODH* as they both functionally converge on the dopaminergic system [66,168] and give rise to a hyper-dopaminergic state that may predispose to psychosis and schizophrenia [66]. Initial evidence of such an interaction came from a transcriptome analysis of a study of PRODH-deficient mice [66,169] showing the up-regulation of *COMT* and thus suggesting the possible up-regulation of the enzyme in order to compensate for the *PRODH* deficiency. 

The mechanism by which *PRODH* deficiency leads to an over-activated dopaminergic system is still an important unknown, but one intriguing hypothesis is that it is due to dysregulated astroglial control of dopamine homeostasis. According to a recent transcriptome analysis of isolated astrocytes [146,148,170] contain all the genes deleted in 22q11DS to some extent or another, and *PRODH* seems to be one among the 30 genes that are highly expressed by astrocytes [146,148,170], thus suggesting that the PRODH enzyme plays an important role in regulating some astroglial functions. Although this role has not yet been investigated in detail, several studies have shown that it is involved in many cell functions other than proline catabolism, including providing energy, shuttling redox potentials between cell compartments, and producing reactive oxygen species [171]. Proline oxidation is an excellent source of energy and the oxidation of one molecule of proline can yield 30 ATP equivalents [171], thus suggesting that PRODH deficiency may cause an equivalent deficit in cell energy. 

In line with the hypothesis that PRODH contributes to cell energy demand, it is highly expressed during post-natal development [172], when the brain grows to approximately 70% of its adult size and brain tissues undergo extensive remodelling. Interestingly, this post-natal growth is due to a rapid increase in the number of neuropil and glial cells, particularly astrocytes. During their post-natal maturation, astrocytes undergo extensive morphological and functional changes [132,159], begin to express the determinants of dopamine uptake, storage, and metabolism, while becoming competent in regulating dopamine homeostasis [150]. Over the last ten years, many studies have shown that defective astrocytic maturation has profound effects on developmental synaptogenesis and circuit formation and function [173,174] and any disruption in astrocyte maturation can be expected to confound the construction and functional architecture of neural networks significantly [175]. The mechanisms regulating post-natal astrocyte maturation have been extensively investigated [176], and the findings of a very recent study suggest that mitochondria biogenesis may play a role in the cellular processes regulating morphogenesis [177]. Developing astrocytes contain a highly interconnected network of mitochondria, but the conditional deletion of the metabolic regulator PPARγ co-activator 1α (PGC-1α) and the consequent interference with mitochondrial biogenesis impairs their morphological maturation and decreases the formation and function of excitatory synapses in the PFC [177], thus underlining the importance of mitochondria to post-natal astrocyte development. The PGC-1α in developing astrocytes can be modulated by the expression of metabotropic glutamate receptor 5 (mGluR5), one of the well characterized signalling ways in astrocytes that has been shown to be highly expressed in developing astrocytes [178,179]. Therefore, the temporal relationship between post-natal maturation of astrocytes and synapses suggests the existence of bidirectional interactions based on the release of glutamate that orchestrate the maturation of functional circuits [132,180]. The mechanism by which altered mitochondrial biogenesis in developing astrocytes affects the formation and maturation of synapses is not known but, as it has been suggested that mitochondrial impairments are also involved in 22q11DS, these findings offer an astrocyte-based mechanism of neural pathology. It is possible that the PRODH and/or mitochondrial deficiency caused by the microdeletion impairs the post-natal maturation of astrocytes and consequently causes the decrease in synaptogenesis and the neuronal cell dysmorphogenesis associated with the syndrome. It is also tempting to speculate that the lack of post-natal maturation somehow affects the way in which astrocytes acquire their dopaminergic competence and therefore gives rise to the hyperdopaminergic state and dysregulated synaptic plasticity associated with 22q11DS.

## 5. Conclusions and Future Directions

Studies in 22q11DS mouse models have revealed neural circuit dysfunctions and a variety of synaptic plasticity alterations that may be responsible for the cognitive impairments observed in the syndrome. Given the critical role of synaptic plasticity in sculpting and in passaging information within neural circuits, network alterations arise because neuronal structure and synapse formation are affected. To investigate these features of the 22q11DS, different studies have focused on hippocampal and cortical neurons, both in vivo and in vitro.

Changes in synaptic transmission, strength, and number of synapses and spines, as well as changes in neuronal excitability have been observed extensively in 22q11DS mouse models. Taken together, all recent findings confirm that synaptic plasticity is involved in the pathophysiological mechanisms of 22q11DS.

Because active synaptogenesis occurs straight after birth and coincides with the increased expression of specific mitochondrial proteins, which are deleted in the syndrome, the possibility of a direct link between mitochondrial metabolism and neuronal function and synapse formation is almost certain. The relationship between mitochondria and neuronal maturation is incredibly tight; indeed, not only the behaviour of these organelles is shaped by development, but they themselves appear to regulate different stages of neurogenesis. Indeed, their importance has been pointed out in different neuronal studies and also recently in glial cells. In fact, we propose a possible role of astrocytes in the pathophysiology of 22q11DS, because of their active participation in synapse formation and function. Mitochondrial impairment in astrocytes lead to important dysfunctions in neurons, therefore their importance is further supported. 

Indeed, as a result of the complexity of the syndrome and because of the need for new therapeutical strategies and targets, mitochondria seem to be a good potential target.

Individuals suffering from 22q11DS present an important decrease in their quality of life, mostly because of their psychiatric manifestations. From a therapeutic point of view, not much has been explored, not only because of the complexity of the developing brain and the hard task of reaching specific brain structures solely with a pharmacological product, but also mainly because of the phenotypic variability of the syndrome. Considerable research is still needed in order to learn about the molecular aspects of this condition and find a potential molecular mechanism to target the treatment of the disease.

## 6. Strengths and limitations and Search Strategy

The main asset of the present review is to provide new insights on how synaptic plasticity disturbances described in 22q11DS may be related to abnormal maturation of astrocytes because of mitochondrial defects, making such disturbances a new potential target for treating psychiatric and cognitive problems associated with this syndrome. To avoid complexifying this assumption, we voluntary restricted our analysis to studies not dealing with in-depth mechanistic information, but simply gave an overview of the most recent findings regarding 22q11DS and the involvement of synaptic plasticity deficits in the occurrence of cognitive abnormalities, and then to link it to developing astrocytes and mitochondria disturbances. In addition, even though the findings reported in the present review originate from animal studies, they have the merit of pointing out the importance of implementing translational studies by using, for instance, hiPSC [181] or the cutting-edge technology of human cerebral organoids that has recently been shown to be suitable to study synaptic processes [182].

## 7. Search Strategy and Selection Criteria

Data for this review were identified by searches of PubMed mainly focused over the last 20 years. References from relevant articles were obtained using the search terms (i) mitochondria or mitochondrial biogenesis; (ii) 22q11 DS or 22q11 deletion syndrome; (iii) schizophrenia or psychosis; (iv) developmental disorders or neuropsychiatric disorders; (v) cognition deficits; (vi) astrocytes, and (vii) synaptic plasticity. In addition, we paid particular attention to use both historical and the most up-to-date references to respectively address well-known assertions and the more recent and pioneering works.
